# Ubiquitous Connected Train Based on Train-to-Ground and Intra-Wagon Communications Capable of Providing on Trip Customized Digital Services for Passengers

**DOI:** 10.3390/s140508003

**Published:** 2014-05-05

**Authors:** Itziar Salaberria, Asier Perallos, Leire Azpilicueta, Francisco Falcone, Roberto Carballedo, Ignacio Angulo, Pilar Elejoste, Alfonso Bahillo, José Javier Astrain, Jesús Villadangos

**Affiliations:** 1 Deusto Institute of Technology (DeustoTech), University of Deusto, 48007 Bilbao, Spain; E-Mails: itziar.salaberria@deusto.es (I.S.); roberto.carballedo@deusto.es (R.C.); ignacio.angulo@deusto.es (I.A.); pilar.elejoste@deusto.es (P.E.); alfonso.bahillo@deusto.es (A.B.); 2 Electrical and Electronic Engineering Department, Universidad Pública de Navarra, 31006 Pamplona, Spain; E-Mails: leyre.azpilicueta@unavarra.es (L.A.); francisco.falcone@unavarra.es (F.F.); 3 Mathematics and Computer Engineering Department, Universidad Pública de Navarra, 31006 Pamplona, Spain; E-Mails: josej.astrain@unavarra.es (J.J.A.); jesusv@unavarra.es (J.V.)

**Keywords:** smart train, ubiquitous communications, Bluetooth piconet network, train-to-ground communication, context-aware services

## Abstract

During the last years, the application of different wireless technologies has been explored in order to enable Internet connectivity from vehicles. In addition, the widespread adoption of smartphones by citizens represents a great opportunity to integrate such nomadic devices inside vehicles in order to provide new and personalized on trip services for passengers. In this paper, a proposal of communication architecture to provide the ubiquitous connectivity needed to enhance the smart train concept is presented and preliminarily tested. It combines an intra-wagon communication system based on nomadic devices connected through a Bluetooth Piconet Network with a highly innovative train-to-ground communication system. In order to validate this communication solution, several tests and simulations have been performed and their results are described in this paper.

## 1. Introduction

The companies that offer transportation services are increasingly interested in enabling remote communications with their fleet by using wireless technologies. These connectivity systems allow them to improve their daily work (by means of new services like fleet management, operation monitoring, safety related applications, vehicle maintenance or/and diagnostics, and so on) and the services provided to the transportation service users.

On the other hand, the widespread use of wireless and Internet technologies in transport systems enables the provision of a large number of new intelligent services. Moreover, the presence of ubiquitous connected vehicles (trains, subways, buses or cars), with inter- and intra-vehicular communication capabilities, as well as continuous connectivity with their control centers, is a key factor to take into account for the new generations of Intelligent Transportations Systems (ITS).

In the railway industry, the sector where our work is focused on, wired networks (such as Ethernet) are commonly used for intra-train communications. These networks are mainly used by the safety and control systems hosted inside a train. The innovation of this work is to enhance the concept of ubiquitous connected smart train by contributing with advances in train-to-ground wireless communication systems [1] and taking advantage of the communication and interaction possibilities of smartphones for communications inside the train. The combination of these two challenges, an intra-train communication system based on nomadic devices and a highly innovative train-to-ground communication system, will be capable of improving user experience of passengers who could enjoy more customized information.

The paper is organized as follows: first, a brief overview of related work is introduced. Second, the current network architecture of the train, where our proposed solution is being tested, is described. Third, the train-to-ground communications design and tests results are exposed. Fourth, intra-wagon communications are proposed, including several simulation results. Finally, the conclusions and future work are expounded.

## 2. Related Work

Mobile devices´ capabilities are constantly improving, allowing Internet access through different technologies (GPRS/UMTS, WiFi, *etc.*). From a vehicular perspective, the goal is to achieve continuous Internet connectivity with their control centers. In [2] it is assumed that in a case study where the trains go from a coverage area of one access technology to another, the combination of several wireless technologies is necessary to achieve such ubiquity.

Consequently, during the last years the application of different wireless technologies has been explored in order to enable non-interrupted Internet connectivity from the vehicles (GPRS/UMTS, WiFi, WiMAX, *etc.*) [3,4]. Hence, hybrid solutions to maintain the vehicle always connected through the best available network link selection have been proposed. Thus, several works related to study the proportion of uninterrupted continuous Internet connectivity on moving vehicles in railway field [5–8] can be found. In this sense, it is considered that the radio frequency based communications are a promising alternative to fixed wireless local networks, such as WiFi. However, its effectiveness is constrained when accessed from moving vehicles. This paper presents a solution that enables and integrates ubiquitous train wagon communication and train-to-ground communication. In the following subsections we describe related work in these two domains.

### 2.1. Train-to-Ground Communications

Nowadays the use of wireless and Internet technologies is increasing in the railway industry enabling bidirectional train-to-ground communications [9]. However, these kinds of communication links applied to this environment have to respond to several challenges related to aspects like coverage, bandwidth, communication disruptions, multiple network interfaces for communications and different priorities in the data transmission, responding at the same time to Quality of Service (QoS) [10] demanded by applications.

There are multiple works regarding communications optimization, including traffic prioritization and QoS control. However, these works are usually focused on networks instead of applications or services that use these networks [11]. In addition, there are industrial solutions designed to respond to these detected communications needs and challenges in transportation systems [1,12]. But neither of these projects establishes a communication system that prioritize data transmissions dynamically, making at the same time a QoS control based on bandwidth availability. The solution proposed on this paper includes a train-to-ground communication system designed to respond to all these challenges.

With the purpose of achieving QoS requirements demanded by services, several communication management and prioritization heuristics [13,14] and mechanism exist [15–17]. Although existing solutions are mainly focused on network aspects and not in final applications and services, other approaches are focused on optimizing the use of the network technologies according to the type of traffic generated by applications (QoS control). Therefore, there is an open research field that can be tackled from two complementary points of view: (1) QoS requirements management which involves technology concepts related to the information to transmit, and (2) aspects about network conditions that make possible the transmission of that information (bandwidth, coverage, latency, *etc.*).

In addition, many of these solutions are focused on mobile environments and are able to monitor network parameters (like bandwidth). The main idea in these solutions is to prioritize communications services allowing or denying communications, or readjusting its data rates, in accordance with QoS requirements demanded by the communication requests and the available networks bandwidth limitations.

These mentioned solutions, applied to transportation, would allow prioritizing vehicle-to-ground wireless communications taking into account its QoS restrictions. However, they are mostly oriented to regulate wireless stations' communications and not final individual applications communication requests. Moreover, they do not monitor previous performance aspects (variable) not allowing to the system to dynamically adjust its performance for more efficiency.

These questions open an interesting line of work to develop adaptive algorithms and interactive control methods that perform this adjustment dynamically. The basic idea is to monitor network conditions in real-time, receive feedback measures of the variables of interest, and based on these measures and QoS requirements, make a wireless communication prioritization assigning to the services the considered bandwidth data rate.

Regarding industrial solutions and projects, the work presented in this paper has considered wireless communications systems which have been widely applied to railway industry in order to respond to the communication needs demanded by railway companies. In this sense, the Safe Driver Machine Interface (SAFEDMI) [18] is a European project which aims to prove the feasibility of using wireless communications links in scenarios related to software update for configuration of train-board systems and data downloads for diagnosis operations.

The railway company EuskoTren (from the Basque Country in the north of Spain) has invested in the renovation of its trains and they have also been involved in technological innovation in order to incorporate new security and reliability systems. This technological innovation includes the definition of a wireless connectivity architecture [19] that enables a train-to-ground communication channel between train systems and railway company control center.

On the other hand, Onboard Wireless Secured Video Surveillance (BOSS) [12] is a European project corresponding to the Celtic/Eureka program. The aim of this project was to develop and validate a reliable broadband communication system between trains and the companies control center with QoS guaranties.

### 2.2. Intra-Wagon Communications

Related to intra-wagon communications, this paper studies the application of Bluetooth piconet networks for intra-train communications. Piconet [20] is a general-purpose, low-power *ad hoc* radio network that provides a base level of connectivity to even the simplest of sensing and computing objects. It provides a broad range of mobile and embedded computing objects with the ability to exploit context aware and connected environment within its close surroundings.

Sensors can use piconet to relay information about the state of the local environment or of a particular device. Personal connectivity is improved because the multitude of mobile and fixed devices commonly used by an individual can be connected by piconet; it might be used to personalize things nearby or allow two devices near each other to interoperate. Embedded networking is also suitable for smart information services: active diaries, alarms, information points, and electronic business cards, for example. The inherent close range connectivity that the piconet provides enables these applications can be context-aware [21].

#### Applications of Interest

Within the railway environment, and leveraging the features of wireless communications and personal smartphones, many applications of interest to railway users can be developed. Some of them concern trip monitoring, others concern location- and located-based services, close communications among passengers, notification and alert messages, registration, ticket booking and payment, intermodal passenger assistance, personal navigation, distribution and exchange of railway-related information, intelligent train control, demand-oriented transportation planning and rescheduling [22], and even more. Indoor positioning using Bluetooth networks has been addressed in many works ([23–25]), but it has so far treated briefly in complex environments such as rail and suburban transport systems [26].

One needs to accurately know the performance of the communication channel to be used since these determine the feasibility of the services provided on it. In such way, Bluetooth is a suitable technology for alarm and event notification, and short messaging. It performs well at small and discrete data transfers, where data, which can be read at any time by a client, can be triggered by local events or user requests. Typical applications inform the user about the conditions on board (temperature, humidity, vehicle speed, vehicle occupancy, *etc.*), about the travel evolution (geographic location, estimated time of arrival at destination, time delay/advance on the scheduled time, *etc.*) or about entertainment (video and audio channels, internet access, *etc.*). But they also allow messaging communication with other travelers, for example to share a taxi on arrival at the destination station, and hiring on board services (cafeteria or media) or at the destination station (taxi, metro ticket, *etc.*).

## 3. System Architecture

Once the different approaches and existing solutions are analyzed and taking an overview of the related work in this area, the proposed solution establishes a communication system that enables intra-train and train-to-ground connectivity. This work has been deployed in a train manufactured by CAF (one of the largest train manufacturers in the world). Concerning integration issues, trains connectivity architecture is one of the most important aspects of the work. Specifically, the train used to carry out our tests has two networks that connect all devices that are deployed on the train.

On the one hand, it is the control network known as Train Communication Network (TCN). This network was the result of the work of the most important railway manufacturers (mainly Bombardier and Siemens) and its architecture is based on IEC 61375 standard [27]. TCN is used to control and exchange of information among the most important elements of the train; basically those responsible for the movement and braking.

On the other hand, there is the Added-Value Network (AVN). This network architecture is very similar to a local area network. It is usually based on the Ethernet standard. The objective of this second network is the connection of the devices that support other essential components of the train, such as: people counting systems, air conditioning systems, infotainment systems. Similar architectures have already been successfully employed in other scenarios, such as intelligent lighting systems and agricultural control [28,29].

**Figure 1.**
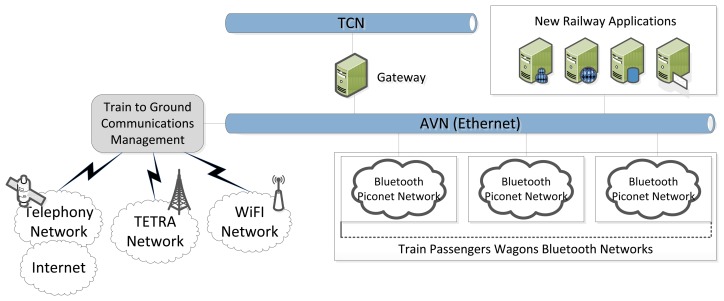
Network architecture of the ubiquitous connected train.

Taking into account this train network architecture (Figure 1), firstly, the solution presented in this paper proposes the creation of a Bluetooth Piconet Network (BPN) [30] inside each passenger wagon. These BPN enable to share and distribute information and contents with passengers' devices, allowing the establishment of ad-hoc operation between dynamic users, which can change along a variable time-span within the proposed scenario. Individual dongles could also be employed, which in principle would not modify the results in radioelectric terms. Secondly, in our approach a reliable train-to-ground communication system is developed which integrates the on-board communication network, GSM radio links, TETRA network and Internet technologies. It becomes the key element to offer ubiquitous remote access to on-board equipment and distribute applications from transportation ground systems.

So, this solution deploys a BPN on each wagon. BPNs are interconnected each other through a train Ethernet network (AVN), which enables an information channel along the train. AVN is also integrated with the train-to-ground communication system in order to achieve external connectivity.

This approach will allow the railways companies to exchange information with their trains and distribute contents and information to the users. Thus, this kind of communications enables the development of new on trip personalized digital services for passengers (e.g., trip information, weather forecast or train connections in destination).

## 4. Train-to-Ground Communications Middleware

In order to respond to train-to-ground communications challenges, we propose a communication middleware that aims to enable several physical network links between train and ground system (3G, WiFi, *etc.*). It chooses the network link considered as the best at every moment according to the bandwidth availability.

Focusing on an application layer middleware it is a more flexible approach being able to introduce new parameters or factors that can be managed to improve communications performance. So, the objective is to develop a dynamic and adaptive communications solution based not only on network conditions or applications request priorities. This system is based also on system historical performance parameters (previous bandwidth values, time in network coverage areas during the transportation routes, previous applications communications performance, *etc.*).

### 4.1. Addressed Requirements

This middleware has been designed to respond to several requirements:

1. *Dynamic and efficient communication request management:* this system prioritizes train-to-ground communications requests taking into account communication urgency criteria, as well as previous performance logs. In addition, the variability of the connectivity conditions influences directly in active communications, demanding a dynamic vehicle-to-ground communication management.
2. *The best bandwidth:* the system always selects the physical link considered as the best taking into account the bandwidth in order to respond to final applications communication requirements. Multiple network link availability maximizes continuous communication capacity, being network active link changes not perceived by final applications. So, this middleware is designed to perform active network link changes avoiding final applications communications disruptions.
3. *Quality of Service:* this solution aims to make a service quality management too. Therefore it is necessary to know the bandwidth availability offered by the network link which is active at every moment, as well as the bandwidth offered by the rest of communications links (although they are not being used). At this point it is essential to establish a set of connection procedures which enable to reserve a certain bandwidth for a particular communication. Therefore, the principal QoS parameter managed by this system is the bandwidth. In this way, the idea is to make bandwidth allocations for different applications communications according to these applications requirements, and allowing them to adjust these allocations depending on the measured available network bandwidth.

### 4.2. Architecture

**Figure 2.**
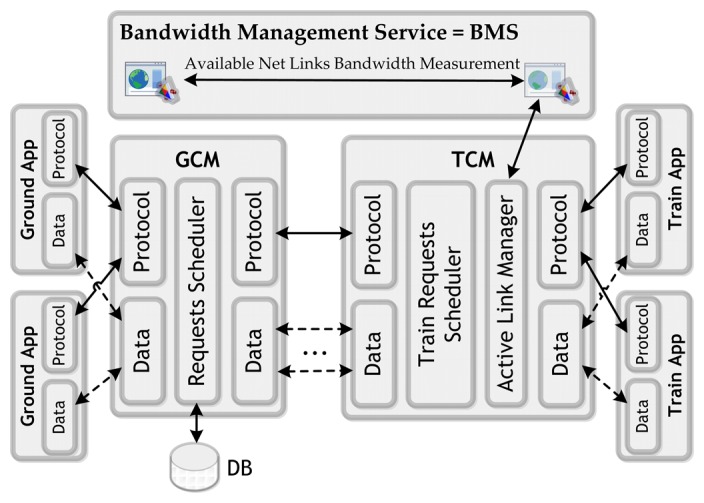
Train-to-ground communication middleware architecture.

This middleware is composed of two software elements (Figure 2); one in the terrestrial side (Ground Communication Manager, GCM), and the other on board the trains (Train Communication Manager, TCM). The former manages the terrestrial aspects of the architecture and the latter the train-side issues. They interact with each other in order to control and manage train-to-ground communications. In addition, this system includes a Bandwidth Measurement Service (BMS) that notifies available links bandwidth values to the GCM at every moment.

In order to establish train-to-ground communications, TCM and GCM can communicate through different communication network physical links. The TCM is who selects the active link considered most favourable for communications based on available link bandwidth measurements notified by the BMS, and then establishes active link connection with the GCM. Two kinds of flows are involved in these communications: data and control. Thus, GCM and TCM on each train communicate each other and exchange commands in order to establish active links and manage the prioritization of train and ground final applications requests. These priorities are managed using specific queue scheduling techniques. The control protocol is defined using XML messages where information is exchanged via TCP/IP sockets. Thus, this middleware is composed of several functional modules:

- *Active Link Selection:* BMS is continuously monitoring the status of all enabled network links, and TCM switch from one to other in two cases: (1) when active link connectivity is lost and (2) when BMS measurements indicate that another link is better than the one established as the active one. In these two cases, the active communication link change without affecting the final applications that do not detect connection interruptions if these link changes occur while they are transmitting. At this point it should be emphasized that the system always defines a single vehicle-to-ground network link as active for communications (the most favourable). So, all communications will always be generated by the channel set as active (WiFi, GSM/GPRS, Tetra, *etc.*) regardless of the availability of other physical channels simultaneously.
- *Request Prioritization:* in order to prioritize communications, the system defines several parameters (variables and statics) that permit it to determine which communications are more critical taking into account connectivity conditions and available bandwidth. Static criteria are parameters that not change over time, and are related to QoS needs and other requirements determined by the final applications characteristics. On the other hand, the prioritization system uses relevant information stored about previous system behaviours. Thus, the system takes into account several dynamic factors (variable over time), being able to readjust the criteria applied in prioritization mechanism permitting to optimize its performance. So, combining these two kinds of criteria, the system calculates a numeric value that represents the fitness of serving a request (priority). Once calculated this value, it is used to discern which communication request is served. Hence, in order to perform requests prioritization, this system develops several queues where requests are sorted by vehicle and priority. Therefore, the idea is that the system reconfigures how it prioritizes the communication requests depending of its behaviour over time, always seeking the most optimal configuration based on system feedback.
- *Vehicle-to-Ground Control Protocol:* GCM and TCM on each vehicle communicate with each other and exchange commands in order to establish active links and manage the prioritization of vehicular and terrestrial final applications communications. The control protocol is defined using XML messages where information is exchanged via TCP/IP sockets.
- *Bandwidth Allocation to Final Applications Requests:* when an application attempts to start a new communication makes a request to the platform, then the system makes a decision about which priority requests can be served concurrently taking into account active link bandwidth limitations, priority parameters and their QoS requirements. Thus, once the system give permission to a communication request, adjust its data transmission rate according to its QoS requirements, the active link bandwidth and the other concurrent active requests. In addition, system could assign more bandwidth than minimally required to a request depending on bandwidth availability and requirements of the rest of active requests.

### 4.3. Validation Tests

All these abilities have being successfully tested through preconfigured scenarios. The tests objective is to evaluate system performance under known conditions which include different kind of final applications with different communications and QoS needs, and changes in different network links connectivity conditions.

#### 4.3.1. Scenario

In a real situation, the vehicle moves from one network coverage area to other, which changes the conditions of the available access networks and bandwidth values. So, in order to set up a scenario as close as possible to real network conditions and bandwidth values, we measured bandwidth of two 3G links of different mobile phone companies along a route.

**Figure 3.**
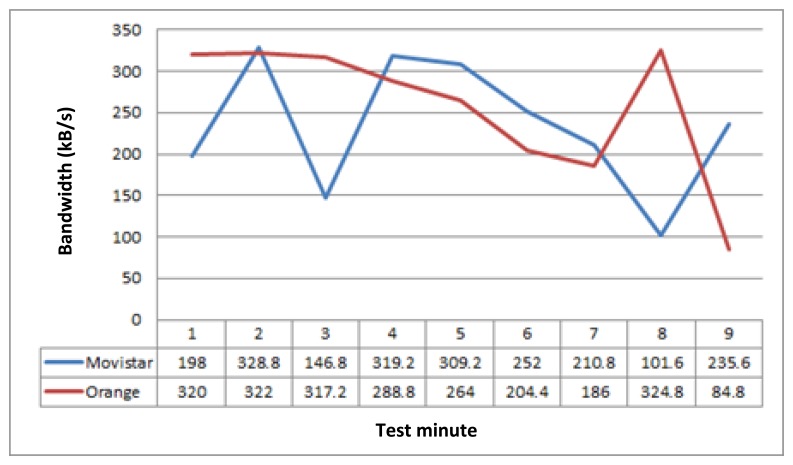
Estimated bandwidth mean values.

Having these values measured, we applied a bandwidth limiter tool (NetLimiter [31]) to simulate these values in our testbed. This external tool enables us to limit the bandwidth of different network links, being able to schedule bandwidth values over time (per minute). So, having measured real values, we estimated bandwidth average (per minute) along the simulated route. Figure 3 shows the bandwidth mean values used in the tests during 9 min.

On the other hand, based on our previous experience working with transportation systems [19,32,33], our communications middleware have been tested by simulating the traffic which is usually generated by applications and services deployed in transportation systems that require vehicle-to-ground communications. Thus, Table 1 shows the selected applications types to perform the tests. This table indicates four kinds of services, their bandwidth requirements (QoS parameters) and the priority levels of transmitted information. Therefore, in order to perform the tests, we have developed a final applications simulator that simulates the network traffic which is usually generated by railway applications.

**Figure 4.**
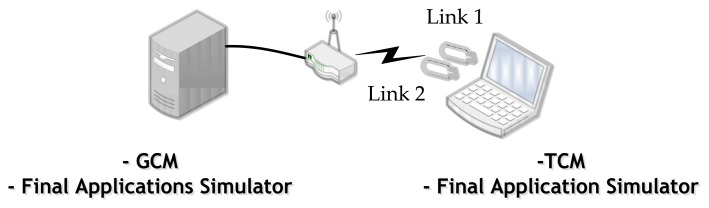
Test set-up.

Based on these bandwidth values and the set of example applications, we deployed the test scenario. It was composed of a laptop for the train system, a PC for the terrestrial system, and a wireless router and two WiFi network links connected to the laptop (Figure 4). Thus, the TCM and a final applications simulator were setup on the laptop, whereas we installed the GCM and also the final applications simulator on the PC. So, the idea is that the vehicle has two different network links to perform communications with the terrestrial side. The bandwidths of these two network links are established by the NetLimiter tool based on the bandwidth measurements mentioned before (Figure 3).

#### 4.3.2. Results

Once the network bandwidth limitations and communication requests of the scheduled applications had been configured, we ran tests in order to evaluate system performance. Tests started launching first the high priority (CCTV in real time) and normal priority (deferred CCTV) requests. Critical requests are related to positioning service. So, this kind of request was created every 10 s, similarly to a real situation. In addition, during the tests, in the second minute was scheduled the launch of the low priority request (vehicular applications log downloads).

**Figure 5.**
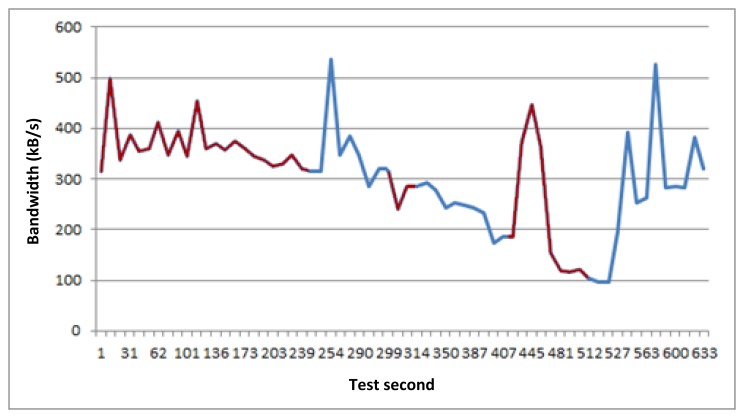
Bandwidth of the active link.

Figure 5 represents the bandwidth values of the available active link for communications notified by TCM to GCM during the tests. Based on this bandwidth values, the middleware manages scheduled communication requests, adjusting the assigned bandwidth to each one taking into account their QoS requirements. In addition, system prioritizes active requests. Thus, if the bandwidth requested for communications is higher than the available bandwidth in the access network selected by the system as active, the middleware selects which requests are more critical, and pauses those that it assumes less critical until conditions change and they can be attended to.

On the other hand, this test has been done through two network interfaces, so Figure 5 not only represents the active link bandwidth values, it also distinguishes the active link changes produced during the tests. Bandwidth values related to link 1 (Orange) are represented with red color, while those related with link 2 (Movistar) are represented using blue color. At this point, it is important to say that these network link changes do not affect to final applications communications. Therefore, when middleware changes the active link, the applications that are transmitting data at this moment do not perceive communication disruptions, so it can be said that these active link changes are not perceived by final applications.

**Figure 6.**
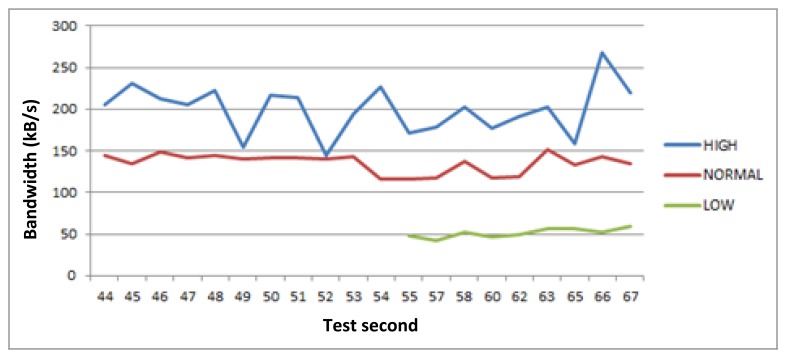
Low priority request launch.

**Figure 7.**
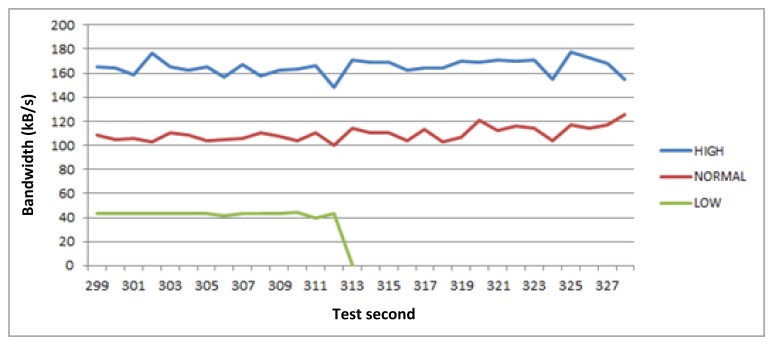
Low priority communication pause for bandwidth limitations.

**Figure 8.**
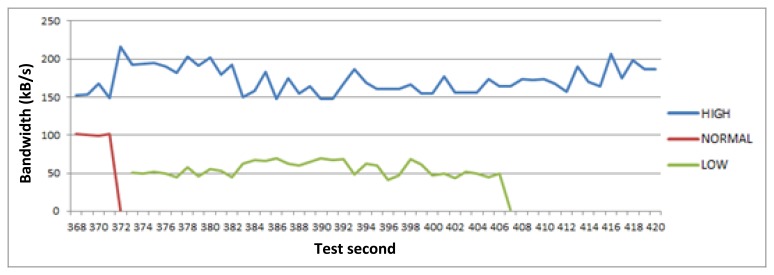
High priority request prioritization.

This test lasts 9 min. If we look at the Figures 5, 6, 7 and 8, the horizontal axis indicates the second of the test. Figure 5 shows the bandwidth values of all active links notified to GCM during the tests. However, Figures 6, 7 and 8 represent three different and descriptive situations managed by the middleware. As can be seen, these graphics do not illustrate critical requests data transmission (positioning service). The reason is that the bandwidth requirement of these requests is very low compared with the other types of services. Thus, the critical requests are not representative and attending to an easier to understand graphic, have not been shown in the results presented here.

Figure 6 shows the moment when the low priority request is launched and admitted to be served by the middleware, starting its data transmission. This kind of request require 40 kB/s of minimum bandwidth to respond to its QoS requirements, so the middleware adjust all active requests bandwidth based on their bandwidth requirements and the active link bandwidth availability. In this case, the global requested bandwidth is lower than the available for communications, so the middleware assigns more bandwidth than the minimum demanded by each request. In addition, in the previous situation, when the low priority request is launched, there were two active transmissions related to high and normal requests. The entry of this new request makes the system readjust the bandwidth assigned to these two requests based on active link bandwidth availability values.

The situation presented in Figure 7 is caused when the available bandwidth of the active link changes and the bandwidth requested by applications is higher than the available bandwidth for communications. Then, the middleware prioritizes the requests and pauses the least priority ones, which cannot be served according to their QoS requirements (minimum bandwidth values, in this case). Therefore, we can observe that in the second 313 of the test the available bandwidth is reduced, so the system stops the low priority request (related to log download service) and readjusts the bandwidth assignation to the other active requests that can continue being served.

Figure 8 shows a representative situation to observe how the middleware always prioritizes the most critical communications when bandwidth values change. In the 368th second of the figure the bandwidth rounds 250 kB/s, and the middleware has paused the least critical request (low priority request). However, a few seconds later the bandwidth availability decreases, and there is no enough to respond to high and normal priority requests' minimum requirements. Thus, the middleware pauses one of them, the normal priority one (deferred CCTV download). However, the bandwidth availability responds to the needs of the high and low priority requests simultaneously, so in this test time interval, there are bandwidth fluctuations that cause the middleware to prioritize communications dynamically adapting their data transfer rate based on available bandwidth, and QoS requirements.

Therefore, the previous functional tests demonstrate that the system performance is successful taking into account its design objectives. The results determine that the middleware improves the performance of the most priority communications, and ensures that all data transmissions will perform in response to the final application QoS requirements.

## 5. Intra-Wagon Communications

Our approach proposes an intra-wagon communications network establishment based on Bluetooth Piconet Networks (BPN), which enable users' ubiquitous interaction with the train information systems. Thus, Bluetooth devices are organized in small networks (piconets) with one device acting as the master and up to seven others acting as active slaves, at any given time [30].

Therefore, the proposed solution applies intra-wagon communications creating a BPN inside each passenger train wagon. The tests have been based on three devices, one master unit and two slaves. The master unit device is placed just below the ceiling in the central part of the wagon, and the two slaves are just above the seats, emulating a real person who is sitting in the wagon sending information with a mobile device.

In addition, simulations have been made using the indoor wagon passenger train as scenario. The wagon has been modeled as a metallic cube, with rows of seats with a polycarbonate base. Simulations are based on the deterministic method of a 3D beam source, with the aid of an in-house developed ray launching code [21,34,35] to analyze the complex scenario of the indoor wagon passenger train. This approach is based in Geometrical Optics and Uniform Geometrical Theory of Diffraction. It is important to emphasize that the topology and morphology of the indoor section of the vehicle have a significant impact in the response of the system. Reflection, refraction and diffraction phenomena have been taken into account, as well as all the material parameters (given by dielectric constant values as well as conductivity values at the operational frequency of the system). The passenger seats are made of polycarbonate, the floors and walls of aluminum and the windows of glass. Simulation parameters are shown in Table 2. The cuboids resolution and the number of reflections have been set to 10 cm and 5 reflections, respectively, to balance accuracy with simulation time.

**Figure 9.**
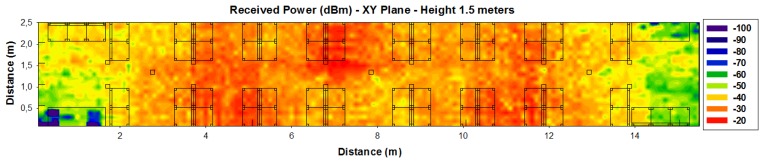
Estimation of received power (dBm) on the indoor passenger wagon train for height of 1.5 m obtained by full 3D Ray Launching algorithm.

Figure 9 shows the power distribution inside the wagon for a height of 1.5 m. As it can be seen, morphology as well as topology of the considered scenario has a noticeable impact on radio wave propagation.

**Figure 10.**
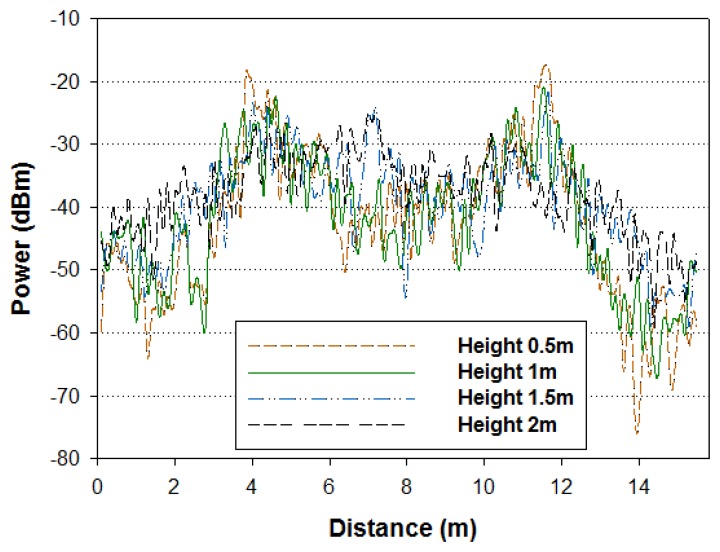
Estimation of radials of received power (dBm) along the X-axis for Y = 1.25 m along the indoor passenger wagon train.

Figure 10 depicts the radials of power along the wagon train (X-axis) for a fixed value of Y, which is Y = 1.25 m, for different heights. It is observed that the distribution of power has a lot of variability due mainly to the strong influence of multipath components.

**Figure 11.**
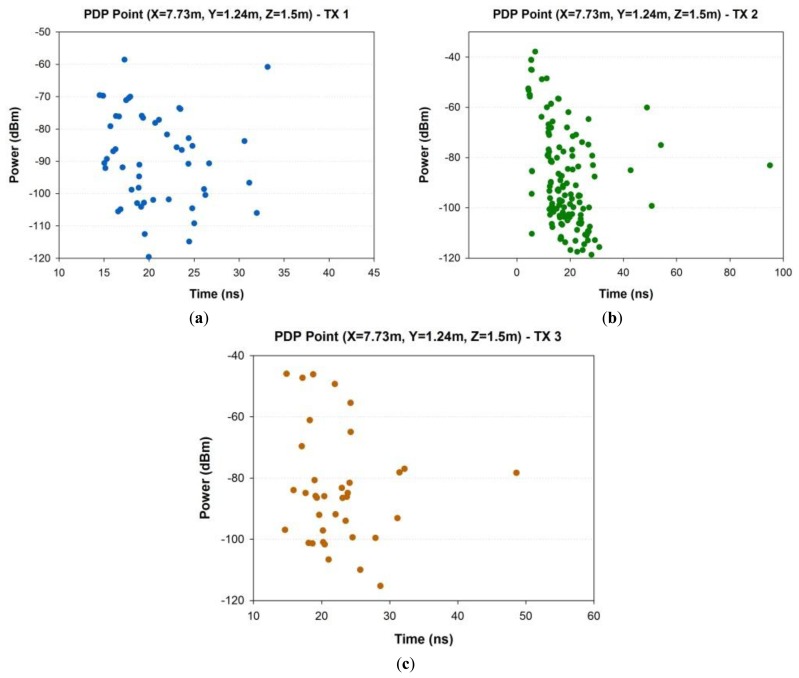
Power Delay Profile at a given cuboid, located at the central location in the indoor wagon train: (**a**) Transmitter 1 (master unit), (**b**) Transmitter 2 (slave 1) and (**c**) Transmitter 3 (slave 2).

As stated above, in this type of environment, the fundamental radio electric phenomenon is described by multipath propagation. To illustrate this fact, the power delay profile for the passenger wagon in a central location has been obtained and is shown in Figure 11 for each transmitter of the BPN. As it is observed, there are a large number of echoes in the scenario due to this behavior of multipath channel.

**Figure 12.**
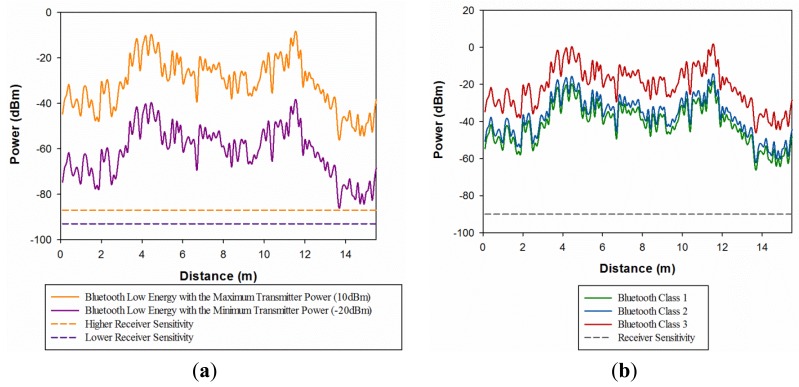
Estimation of radials of received power (dBm) along the X-axis for Y = 1.25 m and Z = 0.8 m along the indoor passenger wagon train (**a**) Comparison between the maximum and minimum transmitter power of Bluetooth Low Energy with the higher and lower receiver sensitivity (**b**) Comparison between classic Bluetooth and receiver sensitivity.

The intra-wagon communication system based on nomadic devices has been assessed for Bluetooth Low Energy (BLE) and classic Bluetooth. BLE is an emerging wireless technology developed by the Bluetooth Special Interest Group (SIG) for short-range communication. In contrast with classic Bluetooth, BLE has been designed as a low-power solution for control and monitoring applications. BLE is the distinctive feature of the Bluetooth 4.0 specification [30]. Figure 12 shows the radials of received power along the wagon train (X-axis) for a fixed value of Y, which is Y = 1.25 m, for 0.8 m height, emulating a sitting person with a Bluetooth device. Figure 12a shows the comparison between BLE with the maximum and minimum transmitter power and the higher and lower receiver sensitivity. It can be seen that even the minimum transmitter power device of BLE has higher values along all the distance of the wagon than the higher receiver sensitivity for this particular complex environment. Besides, Figure 12b shows the comparison between Class 1, Class 2 and Class 3 of classic Bluetooth and all values of the received power for the spatial line considered have higher values than the receiver sensitivity. It must be pointed out that the data of BLE and classic Bluetooth has been obtained from the literature [36].

### 5.1. Measurement Results

**Figure 13.**
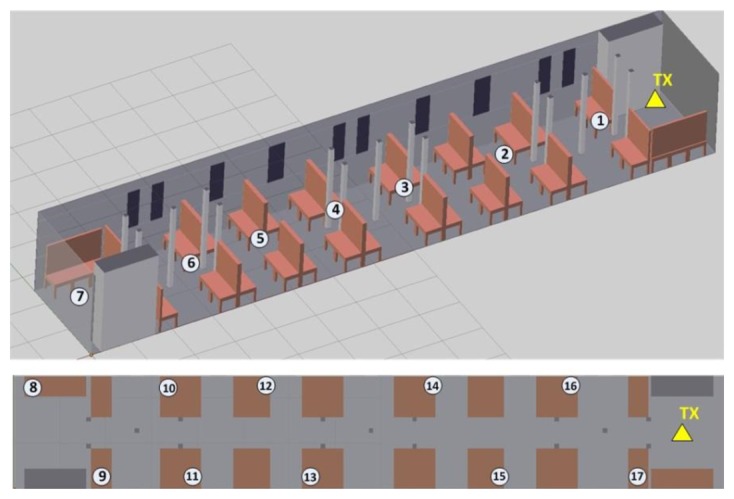
Measurement points within the passenger wagon train.

In order to validate the results previously obtained, measurements in a real wagon train have been developed. A transmitter antenna, connected to a signal generator at 2.4 GHz has been located at the coordinates (X = 14.8 m, Y = 1.25 m, Z = 1.1 m) which correspond with the final part of the wagon train, with a transmission power of −10 dBm. The employed signal generator is a portable Agilent N1996A and the spectrum analyzer is an Agilent N9912 Field Fox. The antennas used are ECOM5-2400 from RS, both omnidirectional antennas. Figure 13 shows the location for the transmitter in the model created for the passenger wagon train and the location of the measurement points. In the central line, measurement points have been taken at a height of 1.10 m. For the lateral measurement points, the receiver location was just above the seats.

**Figure 14.**
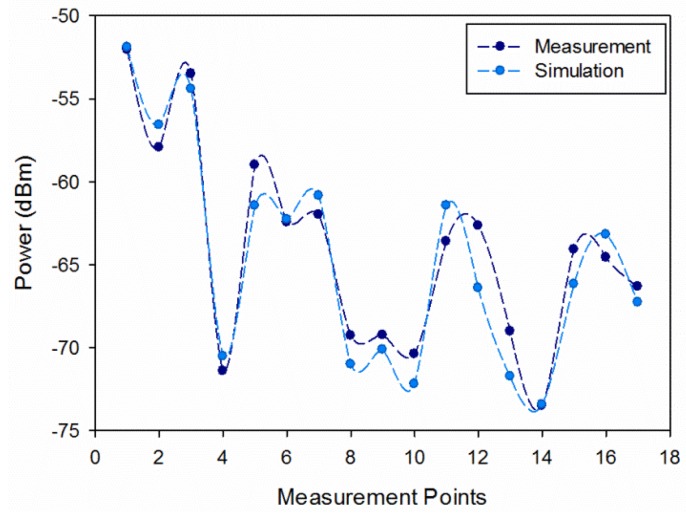
Comparison simulation *versus* measurements for 2.4 GHz frequency in the indoor passenger wagon train.

Figure 14 shows the comparison between simulation and measurement results for the measurements taken along the passenger wagon train. Measurements were performed with 100 MHz bandwidth at 2.4 GHz frequency. The measurement time at each point was 60 s, and the value of received power represented by each point is the higher peak of power shown by the spectrum analyzer for the considered bandwidth (*MaxHold* function in the spectrum analyzer of Agilent). The received power values estimated by simulation have been obtained for the same spatial samples as the real measurements, considering the corresponding cuboid in the three-dimensional mesh of cuboids in which the scenario have been divided. The mean error between simulation and measurements for the indoor passenger wagon train is 1.448 dB with a standard deviation of 0.986 dB. It is shown that it exhibits good agreement and validates previous results.

### 5.2. Rail Service as Proof of Concept

In order to validate the results described above, we have developed RailService, an app developed for Android and IOS mobile devices that allows them to act as a remote control to select the TV channels and volume of the screen embedded in the front seat, which also reveals environmental vehicle parameters (temperature, humidity, speed), the time remaining to reach the destination and an alarm to alert the passenger with a certain advance of the arrival at destination. In order to prevent during screen's degradation due to the continuous, and sometimes inadequate, use of the buttons of the screens (or even the touch screens) we allow the use of the passenger's smartphone as a remote that controls the display and forwards the audio signal to the user. Passengers can then listen to the audio from their own phone and control their screen from their own smartphone.

**Figure 15.**
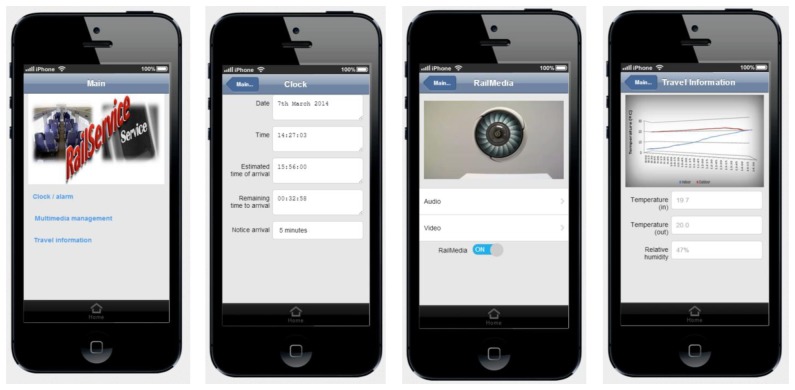
RailService's screen captures.

Figure 15 illustrates the graphic user interface of the related application. We deploy a Raspberry Pi device in each vehicle in charge of collecting and transmitting (via Bluetooth) the information concerning the trip (temperature, location, speed, time to arrival, …) to the RailService App. Similarly, the screen embedded in each seat communicates with the smartphone of the passenger.

**Figure 16.**
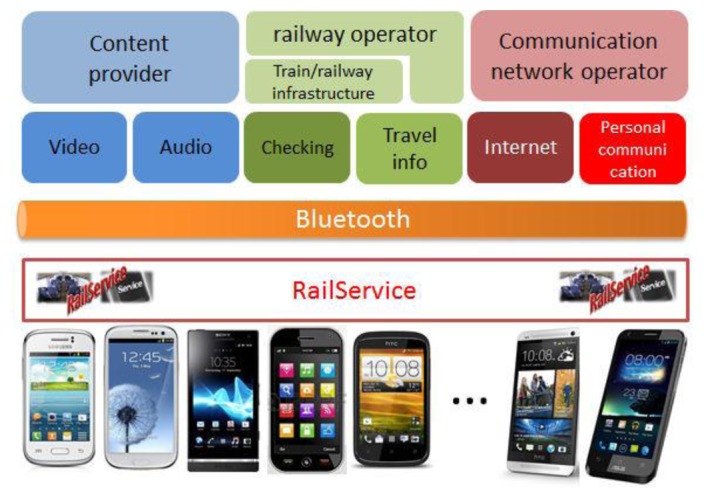
RailService's architecture.

**Figure 17.**
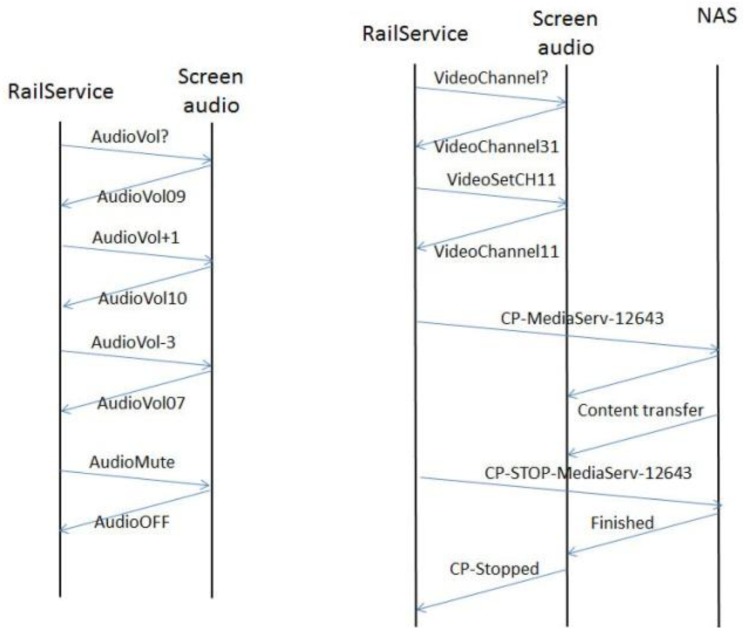
Some communication flows for the remote control of the app.

Audio and video contents are supplied by a content provider (or a set of them). We collect the multimedia contents in a network-attached storage (NAS) in order to avoid the interruption of the information flow when the train traverses areas of low or null coverage that may cause the degradation of the user's perception of the service. The NAS, a Blusens Server Box in our case, makes use of DLNA and UPnP to provide via WiFi the multimedia flow to the screens embedded in the back seats. Figure 16 depicts the architecture of the App. RailService establishes different communication flows (see Figure 17) with both the display hardware and the Raspberry Pi, in charge of collecting and serving environmental information and providing internet access to passengers.

Since Bluetooth was designed to minimize energy consumption, it is not indicated to provide multimedia transfer. For this reason, some authors as [26,37] have worked this issue specific algorithms that allow to dynamically adapting the network to an optimal value of T_poll_ during transmission. But in our case, the replacement of Bluetooth by WiFi technology is not pursued. Our proof of concept is intended to provide greater functionality to the passengers minimizing the potential damage from improper uses of the screens and headphones.

Figure 17 illustrates the protocol of getting the volume of the audio signal, increasing it by a unit, decreasing it by three units, and finally, muting the volume. Furthermore, one can observe the message flow for getting the current channel (31 in this case), selecting channel number 11, selecting the visualization of a certain multimedia content and the finalization of the reproduction.

## 6. Conclusions and Future Work

Transportation companies have been investing heavily in recent years in technological innovation activities in order to improve the quality of services offered to their customers. This is allowing the establishment of wireless communications between vehicles and management facilities of these companies, enabling the development and deployment of new Intelligent Transportation Systems (ITS).

This paper has proposed a solution to provide the ubiquitous connectivity needed to enhance the smart train concept. It includes not only train-to-ground communication systems, but also intra-wagon connectivity which integrates passengers' devices in the environment.

Regarding the last challenge, results of several radioelectric simulations have been presented in order to analyze the viability of the applications of Bluetooth Piconet Networks inside train wagons, using an indoor wagon passenger train as test scenario. On the other hand, a research work focused on the development of a train-to-ground communication middleware designed to respond to communication requirements demanded by railway applications was presented. It manages aspects related to QoS, uses multiple radio and mobile interfaces (GPRS, UMTS, WLAN, *etc.*) and adopts an “always best connected” approach to enhance communications availability and obtain the best bandwidth capabilities, by selecting always the most favorable network link. Finally, future work relative to the exploitation of this communication system by the development of on trip customized added value services for passengers will be explored.

The research area where is focused the research work presented in this paper has still a long way since the progress in telecommunications and its applications for the development of new services in the transportation open a wide range of possibilities (from the point of view of network communications, and also from the point of view of information systems that could be developed around it). In regards to the extension of the presented approach to other transportation systems, as for example suburban systems, the software communication architecture is fully compliance with other systems, but several issues related to physical layer such as bandwidth or channel capacity, should be explored and addressed specifically.

## Figures and Tables

**Table 1.** Set of test applications.

**Service**	**Required Bandwidth**	**Priority**
Positioning	1 kB/s	CRITICAL
CCTV (Real Time)	150 kB/s	HIGH
CCTV (Deferred)	100 kB/s	NORMAL
Vehicular Applicatioón Log Download	40 kB/s	LOW

**Table 2.** Parameters in the ray launching simulation.

**Frequency**		**2.4 GHz**
Vertical plane angle resolution	Δ*θ*	1°
Horizontal plane angle resolution	Δ*φ*	1°
Reflections		5
Transmitter Power		0 dBm
Cuboids resolution		10 cm
